# Germline Variation in Colorectal Risk Loci Does Not Influence Treatment Effect or Survival in Metastatic Colorectal Cancer

**DOI:** 10.1371/journal.pone.0094727

**Published:** 2014-04-11

**Authors:** Hanna K. Sanoff, Lindsay A. Renfro, Pradeep Poonnen, Pratibha Ambadwar, Daniel J. Sargent, Richard M. Goldberg, Howard McLeod

**Affiliations:** 1 Department of Medicine, Division of Hematology/Oncology, The University of North Carolina, Chapel Hill, North Carolina, United States of America; 2 School of Medicine, The University of North Carolina, Chapel Hill, North Carolina, United States of America; 3 DeBartolo Family Personalized Medicine Institute, Division of Population Sciences, Moffitt Cancer Center, Tampa, Florida, United States of America; 4 Division of Biomedical Statistics and Informatics, Mayo Clinic, Rochester, Minnesota, United States of America; 5 Division of Medical Oncology, The Ohio State University, Columbus, Ohio, United States of America; The Chinese University of Hong Kong, Hong Kong

## Abstract

**Background:**

Colorectal cancer (CRC) risk is partly conferred by common, low-penetrance single nucleotide polymorphisms (SNPs). We hypothesized that these SNPs are associated with outcomes in metastatic CRC.

**Methods:**

Six candidate SNPs from 8q24, 10p14, 15q13, 18q21 were investigated for their association with response rate (RR), time to progression (TTP) and overall survival (OS) among 524 patients treated on a phase III clinical trial of first-line chemotherapy for metastatic CRC.

**Results:**

rs10795668 was weakly associated with TTP (p = 0.02), but not RR or OS. No other SNPs carried statistically significant HRs for any of the primary outcomes (RR, TTP or OS).

**Conclusion:**

Common low-penetrance CRC risk SNPs were not associated with outcomes among patients with metastatic CRC.

## Introduction

The survival of patients with metastatic colorectal cancer is improving, with more than half of patients enrolled in recent phase III trials now living more than two years.[1] Despite this improved prognosis, patient outcomes remain heterogeneous. This heterogeneity is increasingly recognized as resulting from distinct molecular subtypes of colorectal cancers,[2] which in turn is influenced by the molecular pathway of carcinogenesis through which cancer develops in individuals.[3] For example the prognosis of patients whose cancers develop through the chromosomal instability pathway differs from those who develop colorectal cancer through germline loss of mismatch repair enzymes, which differs from the poor prognosis of patients with cancers characterized by the CpG island methylator phenotype and BRAF V600E mutation.[4], [5]

Genome-wide association studies (GWAS) have identified a number of loci that increase the risk of developing colorectal cancer. Single nucleotide polymorphisms (SNPs) at these loci, including 8q24, 10p14, 15q13, and 18q21, each confer a small independent increase in the risk of developing colorectal cancer. [6], [7], [8], [9], [10], [11], [12], [13] Given the emerging understanding that the underlying molecular pathway of colorectal carcinogenesis influences prognosis of patients with invasive cancer, one or more of these common “risk” SNPs might be expected to be associated with outcomes of patients with invasive cancer. To examine whether these common low penetrance risk alleles might influence the outcomes of patients with metastatic colorectal cancer, we evaluated the association between candidate risk SNPS and clinical outcomes as measured by radiographic response rate (RR), time to progression (TTP), and overall survival (OS) among colorectal cancer patients treated with first line chemotherapy for metastatic colorectal cancer.

## Methods

### Patients

Germline DNA was available for 524 of the 1694 patients enrolled in North Central Cancer Treatment Group Trial N9741 (registered with ClinicalTrials.gov, NCT00003594), a randomized trial of irinotecan, oxaliplatin and 5-fluoruracil combinations for previously untreated metastatic colorectal cancer.[14] Patients with blood drawn for DNA analysis appeared to be representative of the enrolled population based upon demographic and known prognostic factors.[15] Patients had a median age of 61 years, 95% had an ECOG performance status of 0–1, 86% were White, 8% Black, and 4% were Hispanic (Table 1). The parent study, NCCTG N9741 was approved by the institutional review board at all participating centers prior to patient enrollment. All patients gave written informed consent prior to participation. This secondary analysis of stored specimens and de-identified data was approved by the University of North Carolina IRB (07-0843).

**Table 1:** Characteristics of Study Population.

Characteristics		N = 524 n (%)
Median Age (range)	61 (26–85)	
**Age**	<50	86 (17)
	50–65	255 (49)
	>65	179 (34)
**Sex**	Male	309 (59)
	Female	215 (41)
**Race**	White	450 (87)
	Black	38 (7)
	Other	31 (6)
**ECOG Performance Status**	0–1	500 (95)
	2	24 (5)

### SNP selection

Six candidate SNPs were selected from known common, low penetrance colorectal cancer susceptibility loci identified in early GWAS [8]–[12] or previously reported to be associated with clinical outcomes in patients with established colorectal cancer. Selected SNPs were in minimal linkage disequilibrium with each other to avoid redundancy. All samples were genotyped for each of the candidate SNPs using TaqMan allelic discrimination assays (Applied Biosystems, Foster City, CA, USA) as previously described.[16] Genotyping was performed blinded fashion to clinical data.

### Statistical analysis

The genotype distribution at each locus was examined for deviation from Hardy-Weinberg equilibrium (HWE), stratified by race; none of the SNPs violated the HWE assumption. The distribution of each SNP was evaluated descriptively across key covariates, with no difference in genotype according to age, sex, performance status, or treatment arm. Univariate and multivariate analyses of each individual SNP were then performed to evaluate the association of genotype with response rate (RR), time to progression (TTP) and overall survival (OS). Multivariate models were adjusted for covariates known to affect these primary outcomes in N9741,[17] including age, sex, race, performance status and assigned treatment arm. Given the post hoc nature of this analysis and the multiple SNPs and endpoints assessed, any statistically significant results were to be considered hypothesis generating and to require validation in an independent cohort.

## Results

Among 524 patients enrolled to N9741, there was no significant deviation from Hardy-Weinberg equilibrium for any of the six candidate SNPs, (Table 2) suggesting that while these SNPs are associated with risk of developing colorectal cancer, they may not modify the risk of developing metastatic disease. The six SNPs were investigated for their effects on response rate, time to progression and overall survival. When accounting for multiple testing, there was no association with any SNPs and outcomes of patients with metastatic colorectal cancer (Table 2). The only borderline association was between rs10795668 (at locus 10p14) and TTP. Individuals who were homozygous for the minor allele at rs10795668 (A/A), when compared with individuals who were homozygous for the reference allele (G/G), had a shorter time to progression with an adjusted hazard ratio of 1.43 (95% CI: 1.02–1.99, p = 0.02). No association was seen between this genotype and response rate or overall survival. No other SNPs had a statistically significant association with any of the outcomes of interest (RR, TTP or OS).

**Table 2:** Association of Genotype and Clinical Outcomes of Metastatic Colorectal Cancer Treatment.

		Response Rate		Time to Progression		Overall Survival	
SNP/Genotype	n (%)	Adjusted OR, 95% CI	P value	Adjusted HR, 95% CI	P value	Adjusted HR, 95% CI	P value
**rs6983267 (8q24)**			0.90*		0.51*		0.45*
**T/T**	90 (17)	1		1		1	
**G/T**	245 (47)	0.96 (0.58–1.58)	0.87	1.12 (0.87–1.44)	0.38	1.13 (0.87–1.45)	0.36
**G/G**	189 (36)	1.05 (0.62–1.80)	0.85	1.17 (0.90–1.53)	0.25	1.19 (0.91–1.56)	0.21
**rs7013278 (8q24)**			0.50*		0.29*		0.20*
**C/C**	189 (36)	1		1		1	
**C/T**	239 (46)	1.04 (0.70–1.55)	0.83	1.14 (0.93–1.38)	0.21	1.18 (0.96–1.43)	0.11
**T/T**	96 (18)	1.35 (0.81–2.25)	0.25	0.96 (0.74–1.24)	0.76	0.99 (0.76–1.29)	0.92
**rs7014346 (8q24)**			0.68*		0.21*		0.40*
**G/G**	198 (38)	1		1		1	
**G/A**	230 (43)	0.99 (0.67–1.48)	0.99	1.12 (0.92–1.37)	0.24	1.10 (0.90–1.34)	0.37
**A/A**	91 (17)	1.32 (0.79–2.21)	0.29	0.94 (0.72–1.21)	0.61	0.98 (0.75–1.28)	0.89
**rs10795668 (10p14)**			0.32*		0.02*		0.10*
**G/G**	283 (54)	1		1		1	
**G/A**	198 (38)	1.19 (0.81–1.74)	0.38	0.89 (0.74–1.08)	0.25	0.95 (0.79–1.15)	0.62
**A/A**	43 (8)	0.71 (0.36–1.42)	0.33	1.42 (1.02–1.99)	0.04	1.39 (0.99–1.96)	0.06
**rs4779584 (15q13)**			0.66*		0.87*		0.91*
**C/C**	304 (58)	1		1		1	
**C/T**	180 (34)	1.08 (0.73–1.60)	0.69	0.96 (0.79–1.16)	0.64	1.01 (0.83–1.23)	0.64
**T/T**	40 (8)	1.39 (0.68–2.84)	0.37	0.94 (0.64–1.37)	0.73	1.09 (0.74–1.61)	0.73
**rs4939827(18q21)**			0.30*		0.36*		0.48*
**C/C**	131 (25)	1		1		1	
**C/T**	228 (44)	1.28 (0.81–2.02)	0.29	0.99 (0.79–1.25)	0.96	1.09 (0.86–1.37)	0.96
**T/T**	165 (32)	1.47 (0.90–2.39)	0.12	0.87 (0.68–1.11)	0.25	0.96 (0.75–1.2)	0.25

* overall significance of SNP.

## Discussion

GWAS have identified common genetic variants at multiple loci that increase the risk of developing colorectal cancer. As the underlying molecular pathway of colorectal carcinogenesis influences prognosis of patients with invasive cancer, we used a candidate gene approach to evaluate whether these newly identified risk genotypes might also affect the course of disease following the diagnosis of metastatic colorectal cancer. We found no association between evaluated polymorphisms and clinical outcome of metastatic colorectal cancer.

While these SNPs have been previously well-validated as markers of colorectal cancer risk,[6], [7], [9], [10], [12], [13],[18] the few prior studies have reported variable associations between these low penetrance susceptibility SNPs and colorectal cancer outcomes. In an evaluation of CRC patients of any stage treated at two Chinese hospitals, patients with the risk allele of rs10795668 had a reduced risk of colorectal cancer recurrence, but not overall survival; and the risk allele for rs4779584 was associated with a reduced rate of death.[19] An evaluation of twenty-six SNPs at GWAS-identified CRC susceptibility loci in newly diagnosed stage II and III CRC patients treated with adjuvant fluorouracil-based chemotherapy at MD Anderson found significant associations between multiple SNPs and recurrence (rs10749971, rs961253, rs355527) and survival (rs961253, rs355527, rs4464148, rs6983267, rs10505477).[20] In contrast, two observational cohort studies of colorectal cancer patients failed to find an association between any of these previously cited variants and colorectal cancer outcomes (with the exception of rs10749971 which was not evaluated in either study). [16], [21] One of these, an analysis of incident colorectal cancer cases from North Carolina enrolled in the CanCORS cohort study, found no CRC susceptibility SNP was associated with clinical outcome.[16] The other reported only the minor allele of rs4939827 to be associated with a slight increased risk of death from any stage CRC following diagnosis. [21] A recent study of women with incident colorectal cancer in the Seattle-Puget Sound Cancer Surveillance system, found an association between rs4939827 and rs4464148 and colorectal cancer survival. [22]

The failure of the majority of these SNPs to be validated suggests many of the reported associations are likely chance findings identified in the setting of hypothesis-generating examinations of multiple candidate SNPs. Heterogeneity in patients, cancer stage, and cancer treatment across genotypes may also have confounded the ability of these prior studies to find any small associations. Though the sample for our study is fairly small, it has the advantage of having enrolled a relatively homogenous group with regard to cancer characteristics, treatment, and follow-up, thereby minimizing the effect of these critical confounders on outcomes relevant to metastatic colorectal cancer.

Genome-wide association studies conducted in large patient cohorts with clinical annotation have identified multiple common polymorphisms that confer a small excess risk of developing colorectal cancer. These SNPs help explain the heritability of colorectal cancer beyond the uncommon high penetrance mutations responsible for Lynch and Familial Adenomatous Polyposis syndromes. Our study was underpowered to find small effects of the candidate SNPs on survival, but as prognostic markers with no more than minimal effect sizes are of little clinical value, we believe these results support the notion that these polymorphisms do not warrant further investigation as prognostic markers in advanced colorectal cancer.
